# Regional-Scale Analysis of Antimicrobial Usage in Smallholder Cattle Herds (Aosta Valley, Italy): Why Surveillance Matters

**DOI:** 10.3390/antibiotics13030204

**Published:** 2024-02-22

**Authors:** Federico Scali, Sandra Ganio, Claudio Roullet, Mauro Ruffier, Stefania Bergagna, Giulia Pagliasso, Claudia Romeo, Nicoletta Formenti, Antonio Marco Maisano, Giovanni Santucci, Matteo Tonni, Federica Guadagno, Francesca Mazza, Flavia Guarneri, Giorgio Bontempi, Loredana Candela, Giovanni Loris Alborali

**Affiliations:** 1 Istituto Zooprofilattico Sperimentale della Lombardia e dell’Emilia Romagna, 25124 Brescia, Italy; federico.scali@izsler.it (F.S.); nicoletta.formenti@izsler.it (N.F.); antoniomarco.maisano@izsler.it (A.M.M.); giovanni.santucci@izsler.it (G.S.); matteo.tonni@izsler.it (M.T.); federica.guadagno@izsler.it (F.G.); francesca.mazza@aulss9.veneto.it (F.M.); flavia.guarneri@izsler.it (F.G.); giorgio@bontempi.tech (G.B.); giovanni.alborali@izsler.it (G.L.A.); 2 Azienda USL della Valle d’Aosta, SC Igiene Allevamenti, 11100 Aosta, Italy; sganio@ausl.vda.it (S.G.); croullet@ausl.vda.it (C.R.); 3 Assessorato Sanità, Salute e Politiche Sociali della Valle d’Aosta, Igiene e Sanità Pubblica Veterinaria, 11100 Aosta, Italy; m.ruffier@regione.vda.it; 4 Istituto Zooprofilattico Sperimentale del Piemonte, Liguria e Valle d’Aosta, 25124 Turin, Italy; stefania.bergagna@izsto.it (S.B.); gpagliasso@aslto4.piemonte.it (G.P.); 5 Azienda Sanitaria Locale di Ciriè, Chivasso e Ivrea, 10073 Ciriè, Italy; 6 Globe Institute, University of Copenhagen, 1350 København, Denmark; 7 Ministero della Salute della Repubblica Italiana, 00144 Rome, Italy; l.candela@sanita.it

**Keywords:** antimicrobial stewardship, dairy cows, critical antimicrobials, dual-purpose breed, mountain grazing

## Abstract

Optimising antimicrobial usage (AMU) in livestock is pivotal to counteract the emergence of antimicrobial resistance. We analysed AMU in more than 1000 cattle herds over 11 years (2008–2018) in the Aosta Valley (Italy), a region where 80% of farms house less than 50 cattle. Dairy cows accounted for over 95% of AMU. AMU was estimated using the defined daily dose animal for Italy (DDDAit) per biomass for the whole herd and a treatment incidence 100 (TI_100_) for cows. Average annual herd-level AMU was low, with 3.6 DDDAit/biomass (range: 3.2–4.0) and 1.2 TI_100_ in cows (range: 1.1–1.3). Third and fourth generation cephalosporins, which are critical for human medicine, represented almost 10% of usage, and intramammary antimicrobials accounted for over 60%. We detected significant downward temporal trends in total AMU, as well as a positive relationship with herd size. The magnitude of such effects was small, leaving scant room for further reduction. However, the frequent use of critical antimicrobials and intramammary products should be addressed, following the principles of prudent AMU. Our findings highlight the importance of monitoring AMU even in low-production, smallholding contexts where a low usage is expected, to identify any deficiencies and implement interventions for further AMU optimisation.

## 1. Introduction

Optimising antimicrobial use (AMU) in livestock production is crucial to address antimicrobial resistance (AMR) within the One Health paradigm [1,2,3]. Although steadily decreasing, AMU in Italian animal production is still among the highest in Europe [4]. Monitoring AMU is pivotal to guide and to verify the effectiveness of antimicrobial stewardship (AMS) interventions aimed at reducing and optimising usage [5]. In livestock, AMS encompasses a series of actions aimed at reducing the risk of infectious disease occurrence (biosecurity and management), optimising the treatment regime (dosage, duration and route) and prudent AMU [6,7,8]. The latter involves both the veterinarian in the choice of the appropriate antimicrobial and the farmer in the correct administration of said antimicrobial [6]. Choosing the appropriate drug is particularly relevant, since antimicrobials are not considered of equal importance: international associations, such as the World Health Organisation (WHO) and the European Medicines Agency (EMA), identify specific antimicrobial classes that are critical for human medicine and whose use in the veterinary sector should be limited [9,10]. These antimicrobials can represent life-saving treatments for humans where therapeutic alternatives are limited; therefore, containing the spread of AMR for these classes should be a priority to preserve their efficacy.

Different livestock sectors may contribute differently to a country’s AMU, both in quantitative and qualitative terms (i.e., usage of critical antimicrobials). Currently, detailed information on AMU by production sector is not yet available for Italy. In countries with well-established monitoring systems, such as Denmark and the Netherlands, dairy farming has a lower AMU when compared to other sectors such as pig, poultry or veal calf production with low to no use of critical antimicrobials [11,12]. However, several studies in other countries conversely report a frequent administration of critical classes in dairy cattle [13,14,15,16,17,18]. Additionally, the potential contribution of farm factors, such as herd size, to AMU in this livestock sector is still debated [19] and the role of smallholders needs further clarification. Information on AMU in small Italian cattle herds is limited, with only a couple of studies reporting an estimation of AMU in small or small-to-medium farms, both highlighting low usage [20,21]. Although there is no univocal definition of ‘smallholder’, as its meaning largely depends on production and geographic context [22,23,24,25,26], at European level, the European Food Safety Authority (EFSA) proposed that all herds with less than 75 cows can be considered as smallholders as long as they also fulfil at least two of the following criteria: mainly family-owned, a limited use of concentrates, housing of local breeds and/or specific certified production (e.g., organic, Protected Designation of Origin) [22].

The present study aims therefore to describe and analyse AMU in a regional-scale dataset comprising mostly cattle herds that can be classified as smallholder according to the EFSA definition. In particular, our dataset consists of an 11-year prescription data series from over 1000 herds located in the Aosta Valley, a mountainous region in the Italian Alps. This will allow us to identify possible deficiencies in terms of prudent AMU, aiding future AMS interventions even in a smallholding context, where high AMU is not expected.

## 2. Results

**Figure 1:**
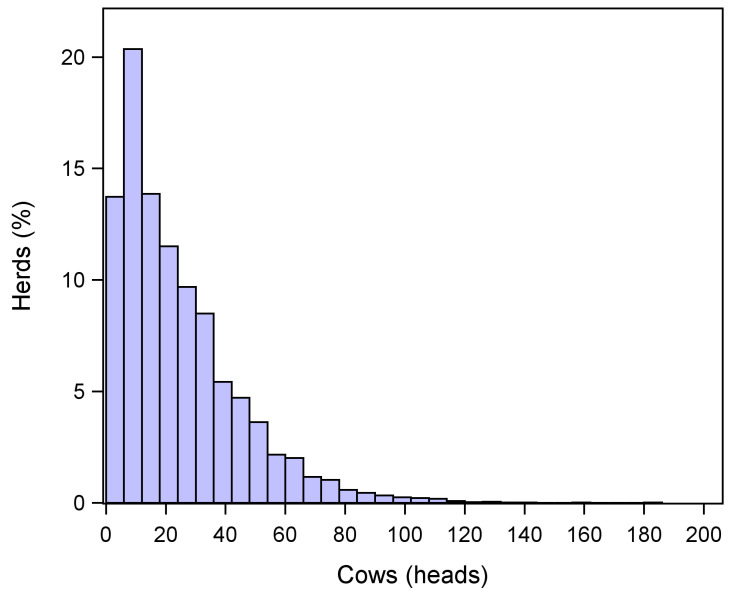
Frequency distribution of cattle herds (*n* = 1260) included in the study by number of housed cows.

A total of 1260 different herds were included in the study; 80.2% of these herds housed fewer than 50 cattle, 16.4% between 50 and 100, and 3.4% more than 100. Regarding cows specifically, 97.2% of the herds housed fewer than 75 heads (Figure 1).

The average number of monitored herds per year was 808 (range: 693–931). This corresponds to a mean of 19,781 cows per year (range: 18,895–20,837), 8344 heifers/beef per year (range: 7633–8895) and 2654 calves per year (2512–2823). Detailed herd data by year are reported in Table 1.

During the study period, the total number of herds decreased significantly (parameter estimate ± SE = −27 ± 1 herds/year; *p* < 0.0001; R^2^ = 0.98), from 931 in 2008 to 693 in 2018. The number of cattle in the region decreased as well (−308 ± 37 heads/year; *p* < 0.0001; R^2^ = 0.87), but less strongly: from 31,635 to 29,040 heads. As a result, the average herd size increased significantly (0.90 ± 0.06 heads/herd/year; *p* < 0.0001; R^2^ = 0.96), from 34.0 heads/herd in 2008 to 41.9 heads/herd in 2018.

Overall, average annual herd-level AMU was 3.61 DDDAit/biomass (range: 3.21–4.00) while usage of WHO’s Highest Priority Critically Important Antimicrobials (HPCIAs) was 0.38 DDDAit/biomass (range: 0.34–0.42). A detailed breakdown of herd-level AMU by year is reported in Table 2.

The five most used classes accounted for 70.3% of the total AMU during the study period. In detail, rifamycins accounted for 17.0% of AMU, aminopenicillins for 15.3%, aminoglycosides for 14.0%, beta-lactamase-resistant penicillins for 13.2%, first- and second-generation cephalosporins for 10.8%. Critical antimicrobials represented 14.5% of total usage, and in particular, third- and fourth-generation cephalosporins accounted for 9.4% of AMU, macrolides for 3.9%, fluoroquinolones for 1.1% and polymyxins for 0.1%.

**Figure 2:**
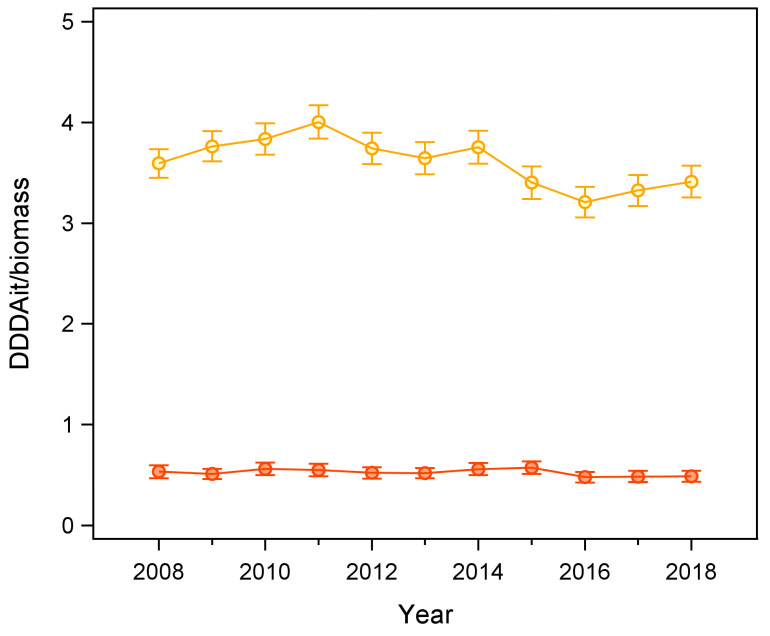
Temporal trend of the average annual antimicrobial usage (AMU) per herd in cattle reared in the Aosta Valley (Italy): total AMU (orange) and usage of critical classes (red) included in the WHO’s Highest Priority Critically Important Antimicrobials list (i.e., polymyxins, quinolones, macrolides, third- and fourth-generation cephalosporins). Means were weighted on standardised biomass, and error bars represent 95% Confidence Intervals.

There was a downward trend in the annual average AMU per herd during the study period (−0.05 ± 0.02 DDDAit/biomass per herd per year; *p* = 0.014; R^2^ = 0.45; Figure 2), while usage of critical antimicrobials remained stationary (*p* = 0.2; Figure 2).

Among the HPCIA classes, only macrolides showed a slight but significant reduction during the study period (−0.006 ± 0.002 DDDAit/biomass per herd per year; *p* = 0.003; R^2^ = 0.60). Detailed data on annual AMU distribution by antimicrobial class are reported in Table 3.

Intramammary products for dry cows were the most used antimicrobials, accounting for 42.8% of the total AMU over the study period, followed by injectables at 34.3%, intramammary for milking cows at 20.4%, intrauterine at 1.3% and oral products at 1.2%. Usage of intramammary antimicrobials for dry cows decreased during the study period (−0.034 ± 0.008 DDDAit/biomass per herd per year; *p* = 0.002; R^2^ = 0.64), from 1.73 DDDAit/biomass per herd in 2008 to 1.45 DDDAit/biomass per herd in 2018.

Overall, 96.4% of all the antimicrobials used during the study period were administered to cows, 2.7% to calves and 0.9% to heifers/beef. Average annual TI_100_ in cows was 1.21 (range: 1.06–1.33). Detailed data on annual AMU in cows are reported in Table 4.

**Figure 3:**
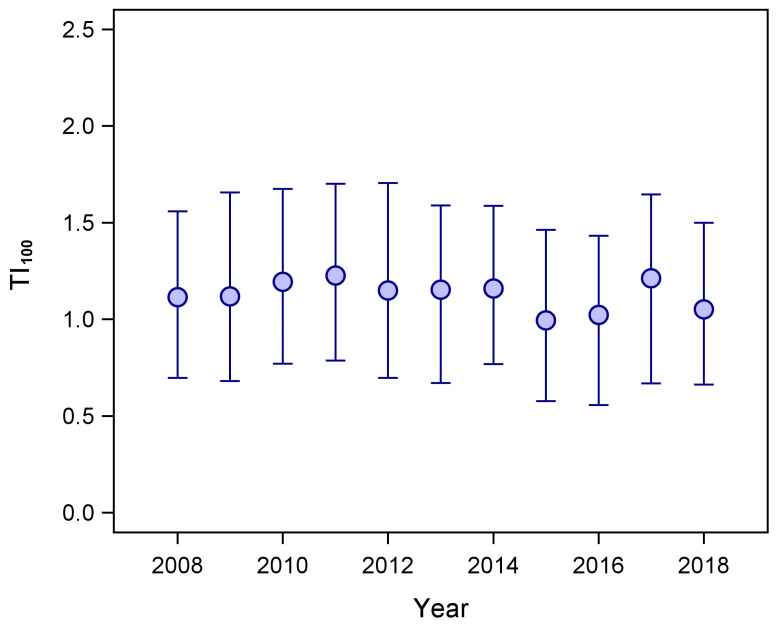
Median antimicrobial usage from 2008 to 2018 in cows housed in Aosta Valley cattle farms, expressed as treatment incidence 100 (TI100). Error bars represent the interquartile range.

Total AMU in cows showed a small but significant decrease over the study period (−0.008 ± 0.001 TI_100_/year; F_1,7621_ = 41.3; *p* < 0.0001; Figure 3) and was positively influenced by herd size (0.0004 ± 0.0002 TI_100_/cow; F_1,7621_ = 5.3; *p* = 0.021).

On any given year, roughly half of the herds in the area employed some critical antimicrobials on cows (min—41.1% in 2008, max—50.5% in 2015). The probability of using critical antimicrobials was positively affected by herd size (χ^2^_1_ = 849.3; *p* < 0.0001): a 10-cows increase led to a 50% increase in the odds of using HPCIAs (OR = 1.50; 95% CI = 1.47–1.54).

## 3. Discussion

We analysed regional-scale AMU data in cattle in the Aosta valley, an area of Northern Italy characterised almost entirely by smallholders, describing the AMU trends in terms of total usage, usage of critical antimicrobials and administration routes over an 11-year period.

To estimate AMU, we have used two different standards, the DDDAit/biomass and the TI_100_, to ensure comparability with previous Italian studies carried out in different bovine production settings [15,27,28]. Indeed, comparison of AMU data collected in different settings is often made difficult by the lack of a common metric and the widespread use of different standards [5,29].

In terms of production, from 2008 to 2018, we observed a progressive reduction in the number of herds in the region and a parallel increase in herd size, which is in line with the national trend [30]. This trend may be due to changes in production and market conditions that favour larger herds, which are potentially more cost-effective. Although the investigated farms are classified as mixed operations, they can be considered akin to dairy farms. Even though a few males are usually kept for meat production, dairy cows make up the bulk of the herds. Even though wide differences among herds were found in terms of overall AMU, more than 95% of antimicrobials were administered to cows. This result suggests that this animal category should be the first target in case of stewardship interventions and confirms the importance of tracking AMU in the animal categories within a herd.

The average herd-level AMU in the area decreased significantly (*p* < 0.0001) over the study period, both when considering the herd as a whole and when considering only cows. However, the effect size was rather small, with an annual average reduction around 1%. Overall, AMU was generally low for the Italian context: the median DDDAit/biomass reached a value of 3.2 in 2018, which is 33% lower than the 4.8 reported in another Italian study on dairy farms carried out using the same standards but in a different geographic area [15]. This result could be explained by the different farming conditions. Indeed, our sample consisted almost entirely of smallholders housing low-yielding dairy cows, while in the other study, larger farms (i.e., with a median number of cows almost seven times higher) housing high-yielding breeds were considered [15]. In cows, the average TI_100_ was 1.2, which is 33–40% lower than the values reported in Italian studies on beef cattle that used the same metrics [27,28]. These results are in contrast with another study in Central Italy [31] that found a higher consumption in dairy cattle than in beef cattle. Nevertheless, in this case, the discrepancies could be explained by the different AMU standards and farming conditions (e.g., larger herds, different climates). A moderately higher average AMU (around 16%) in dairy cattle when compared to non-dairy cattle was also reported in the Netherlands [32]. However, also in this case, the results could be influenced by the different production systems.

Comparing the results of this study with those in other countries is challenging due to the lack of a common standard for estimating AMU in veterinary medicine. Indeed, even DDD-based standards may differ considerably among countries due to different antimicrobial dosages, combination products, standard animal weights, etc. [29,33]. A step towards a more harmonised approach to estimating AMU in livestock was taken by the EMA in 2016, when the agency published the defined daily doses for animals (DDDvet) for cattle, pigs and poultry [34]. However, such units of measurement are still incomplete, as they are not available for dry cow intramammary products [34], which were the most commonly used in the farms involved in this study, and for some other antimicrobials. For example, a study on Italian beef farms found that only 75% of the AMU measured by DDDAit could also be measured by DDDvet [28]. In humans, a common standard has been available and well established for years. In this case, DDDs are assigned by the WHO on the basis of the Anatomical Therapeutic Chemical (ATC) code and the route of administration (https://www.who.int/tools/atc-ddd-toolkit/about-ddd, accessed on 13 February 2024). Although arguably complex and costly, a similar approach could be used in future for the veterinary sector too, ideally managed by the World Organisation for Animal Health (WOAH) assisted by other agencies with expertise on the subject (e.g., the WHO, EMA). A Belgian study on adult cows reported, on average, a higher AMU than the present study (2.1 vs. 1.2) [18]. Although both studies considered very similar standards, the Belgian farms involved were larger, with an average number of cows per herd almost three times higher. Two US studies on even larger herds, one set in Wisconsin and one in Ohio and California, reported, respectively, a higher AMU (33%) [13] and a very similar AMU [35] to that found for the cows included in this study. Nevertheless, the former study considered a higher-risk weight (680 vs. 600 kg) [13] while the latter used a very different standard for intramammary dry products [35], which—leads to a lower estimate of these antimicrobials (about a quarter for the same amount of products administered). A Japanese study reported higher AMU values in cows (3.9) while considering a slightly higher standard weight (635 kg) [36]. However, also in this case, the average herd size was almost 50% larger.

The usage of critical antimicrobials was frequent and generally stable over the study period, with only macrolides showing a limited downward trend. In particular, third- and fourth-generation cephalosporins accounted for almost 10% of the overall AMU. These findings are consistent with several previous studies on dairy farms [13,14,15,16,17,18] and are particularly worrying considering the importance of these antimicrobials for human medicine. Usage of macrolides was relatively frequent as well, representing 4% of total AMU. The categorisation of this antimicrobial class is still under debate: the WHO classifies macrolides as HPCIAs [10], while the EMA considers them a lower-priority class (category C) [9]. One of the prioritisation factors that led to the inclusion of macrolides in the HPCIAs is the emergence of resistant *Campylobacter* spp. from non-human sources [10]. Nevertheless, milk and dairy products seem to be a less important source of *Campylobacter* spp. when compared to other animal food products such as poultry meat [37]. The use of other critical antimicrobials was less concerning, with an infrequent administration of fluoroquinolones (1% of the total AMU) and an almost null consumption of polymyxins (0.1%). Awareness campaigns among farmers and veterinarians could be a first step to address the frequent use of some critical classes, promoting the prescription of critical antimicrobials only in the absence of viable alternatives, as also recommended by the Italian guidelines on prudent AMU in animal production [38], which may have contributed to the general reduction in sales of these classes in Italian livestock occurring in recent years [4].

Mastitis and dry cow therapy are considered the main cause of AMU in dairy production [39,40,41,42,43]. We were unable to obtain information on the reasons for treatment, but intramammary products accounted for more than 60% of the overall AMU. These findings confirm that even in small herds with low production, it is essential to promote mastitis control, udder health and, where possible, selective dry cow therapy. In this context, the downward temporal trend in the usage of intramammary products for dry cows, although limited, may suggest a growing awareness of the matter.

Although statistically significant (*p* = 0.021), the observed relationship between AMU and herd size (i.e., a TI_100_ increase below 0.05 every 100 cows) can be considered negligible, at least in a smallholding context. Studies investigating the relationship between AMU and herd size in dairy production reported contrasting results. A significant positive effect of herd size on AMU was described in Danish [44] and Pennsylvanian [16] farms. This effect was also reported by a recent Canadian study, but it was mostly determined by the use of ionophores [45], which are usually not considered in AMU studies. On the other hand, no such relationship was found in Italy [15], Japan [36] and Argentina [46]. A German study reports an association between AMU and farm size only for dairy calves [47], while another Canadian study reported this relationship only for some specific classes but not for overall AMU [17]. Finally, it should be emphasised that the abovementioned studies were carried out on dairy farms of widely different sizes but in all cases larger than the average herd in the Aosta Valley. Our results suggest that herd size is not necessarily a risk factor for high AMU and that other factors (e.g., farming conditions, low- or high-yielding breeds) may play a relevant role in combination with herd size. The size of the herd also had a significant effect (*p* < 0.0001) on the probability of administering HPCIAs, suggesting that larger herds may be more focused on increasing production and therefore may select what are believed to be the most effective antimicrobials on the market.

Overall, although we observed some downward temporal trends in usage and some associations between AMU and herd size, the magnitude of such effects was rather small because of the generally low AMU. Indeed, this specific production context can be considered low risk for AMU in quantitative terms, with scant room for further improvement. However, the frequent use of critical antimicrobials detected through our analysis was unexpected and deserves further attention.

Although our study included data from a large number of herds and over a long period of time, it has some relevant limitations. First of all, we investigated only a specific geographical area, and although this peculiar cattle production is common to several Alpine regions, our results may not be representative of other smallholding contexts. Furthermore, it was not possible to validate the data with an external source; thus, some of the prescribed antimicrobials may not have been used and we cannot fully rule out data entry errors. In particular, we cannot exclude that some injectable antimicrobials registered on cows were actually used in other animal categories. In addition to multi-year trends and the effect of farm size, it would have been interesting to investigate why the vast majority of antimicrobials were prescribed to cows and if other animal categories were somehow neglected. Finally, other factors that we could not take into account, such as seasonality, pasturing, milk quality and animal welfare, may have influenced the AMU and should be therefore investigated in the future with further ad hoc studies.

## 4. Materials and Methods

### 4.1. Geographical Background

The Aosta Valley is an Italian administrative region located in the northwest of the country, amidst the Alps (Figure 1). With a territory of 3263 square kilometres and 125,666 inhabitants, it is both the smallest and the least populated of the 20 Italian regions. Similarly to other Alpine areas, cattle farming in this mountainous region consists of family-owned smallholder mixed herds, housing a local dual-purpose breed (Valdostana), where dairy cows are the most represented animal category and mountain grazing is practised during the summer. Milk is used primarily for dairy products with a Protected Designation of Origin. Milk production is low, considering a population of around 19,000 cows and a total yearly milk yield of about 28,000 tonnes [48].

### 4.2. Data Sources and Management

In Italy, the national veterinary service is an integral part of public health, and each administrative region manages public health independently within their territorial jurisdiction. This study is the result of the collaboration between the local authorities of the Aosta Valley and the national authorities, represented by the Italian Ministry of Health and the Istituti Zooprofilattici Sperimentali (IZSs). An estimation of AMU was performed for all the herds housing at least two cows. The 2008–2018 data on drugs prescribed on the bovine herds in the Aosta Valley were collected through two channels: (i) data for the 2008–2014 period were digitised by local authorities from paper prescriptions, an activity once included in the regular pharmacosurveillance controls; (ii) subsequent data (2015–2018) were extracted directly from the regional electronic prescription system, established by local authorities a few years before the national one. Over-the-counter antimicrobials for livestock are not available in Italy, and farmers can only purchase them when prescribed by a veterinarian. Some of the WHO’s HPCIAs (third- and fourth-generation cephalosporins, macrolides, fluoroquinolones and polymixins) [10] are registered for use in livestock but recommended only in the absence of viable alternatives [38].

**Figure 4:**
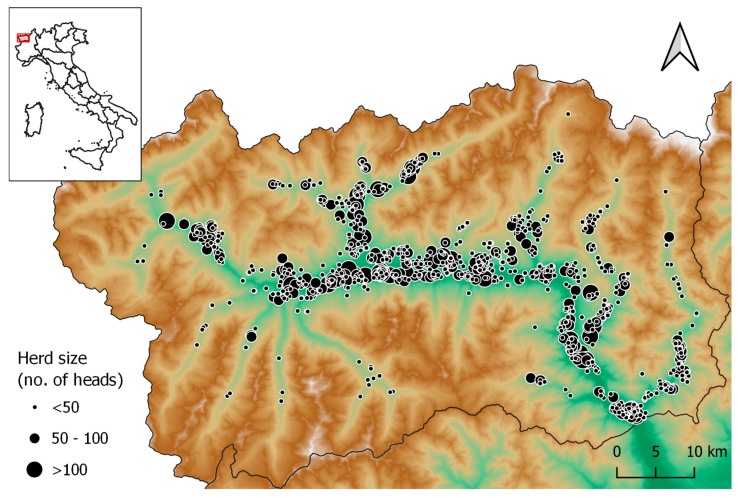
Location of bovine herds (*n* = 1260) within the Aosta Valley region (Northern Italy) for which at least one year of antimicrobial usage data between 2008 and 2018 was available. Dot size is proportional to herd size as defined in the map legend. Map created using QGIS 3.22 software.

Herd data regarding the same period were provided by the local authorities. For every year included in the analyses, complete data were obtained for at least 90% of all the cattle farmed in the region. All these data were imported into the national surveillance system, ClassyFarm (www.ClassyFarm.it, accessed on 19 January 2024), which is owned by the Italian Ministry of Health and managed by IZS della Lombardia e dell’Emilia Romagna. The system began receiving full national data on AMU only during the second half of 2019, when the Italian electronic veterinary prescription system became mandatory. However, ClassyFarm also allows the submission of retrospective data for research purposes: in this case, the AMU is calculated automatically, and the resulting data can be extracted for further analysis. An estimation of AMU was performed by the system, and AMU data were then exported and managed using Microsoft Access and Microsoft Excel (Microsoft Corp., Redmond, WA, USA). The AMU was calculated at the herd level and also, for each herd, at the animal category level, using the ClassyFarm standards for this type of mixed cattle farming: calves (birth to 6 months), heifers/beef (6 to 24 months) and dairy cows. Figure 4 shows the location and size of all the herds included in the study.

### 4.3. Estimation of Antimicrobial Usage

The AMU was estimated at the herd level using the defined daily dose animal for Italy (DDDAit) as a standard metric [15,49]. Briefly, DDDAit represents the standard amount of active ingredient, in milligrams, administered per kg of live weight per day (mg/kg/d), as stated by the summary of the product characteristics. A detailed description of the standard considered in peculiar cases (e.g., range of dosages, antimicrobial combinations, intramammary products, etc.) has been reported in previous studies [15,49].

Annual AMU was calculated for each herd as the total DDDAit per Kg of standardised biomass (DDDAit/biomass) for all active ingredients prescribed and, for each animal category within a herd, as a treatment index 100 (TI_100_).

The DDDAit/biomass standard has been described in detail in a previous study [15] and was calculated according to the following formula [15]:

DDDAit/biomass = (mg of active ingredient prescribed/DDDAit)/(cows × weight at risk + heifers/beef × weight at risk + calves × 2 × weight at risk)(1)

The ‘weight at risk’ for cows, heifers/beef and calves have been set in ClassyFarm at 600, 300 and 100 kg, respectively. Since AMU was considered on an annual basis, and calves include animals up to six months of age, the average number of housed calves was multiplied by two [15].

The TI_100_ was calculated for each year, herd and animal category within the herd (cows, heifers/beef, calves) using the same standards of weight used for DDDAit/biomass, according to the following formula [49,50]:

TI_100_ = [(mg of active ingredient prescribed/DDDAit)/(heads × weight at risk × days at risk)] × 100 (2)

The ‘days at risk’ for cows and heifers were set at 365 days, while for calves, they were set at 180 days. The TI_100_ can be interpreted in three manners [50]: as the percentage spent under treatment by an animal during its production cycle, as the number of days under treatment per 100 days of production, or as the number of animals under treatment every 100 animals housed in the farm on any given day.

Usage of antimicrobials classified as HPCIAs by the WHO (i.e., third- and fourth-generation cephalosporins, macrolides, polymyxins and quinolones) [10] was considered “critical”.

### 4.4. Statistical Analysis

Firstly, we explored the variation in the total number of herds, heads and the average number of heads per herd during the study period through linear regressions. Temporal trends in annual average AMU per herd, usage of HPCIAs (total and by class) per herd and administration routes per herd (all expressed as DDDAit/biomass) were also explored by means of linear regressions. Subsequent analyses were focussed on cows’ data, since 96% of all the antimicrobials and 94% of HPCIAs were prescribed to this animal category. The temporal variation in cows’ AMU at herd level (expressed as log-transformed [ln(x + 1)] TI_100_) was analysed through a mixed linear model, while the probability for a herd of using (y/n) critical antimicrobials was explored through a mixed logistic regression. In both models, year and herd size were included as explanatory variables and herd ID was included as a random term to account for repeated measures of the same herd in different years. The presence of influential observations and the normality of residuals were assessed visually, by means of Cook’s D plots and QQ plots, respectively. Statistical significance was set at α = 0.05. All average AMU data are presented as means weighted on standardised biomass. Statistical analyses were performed in SAS/STAT 9.4 software (SAS Institute Inc., Cary, NC, USA).

## 5. Conclusions

The results of our study highlight the importance of monitoring AMU even in a smallholding, low-production context. Indeed, even though the overall consumption was low, usage of critical antimicrobials was rather common and should be the first target of an awareness campaign. The differences among herds and animal categories in terms of AMU confirm the relevance of tracking usage at different levels, and the frequent administration of intramammary products corroborates the importance of promoting udder health as well as mastitis control. Finally, this work also represents a positive example of cooperation between local and central authorities.

## Figures and Tables

**Table 1:** Annual descriptive statistics of the cattle herds included in the analysis of antimicrobial usage in the Aosta Valley (Italy) from 2008 to 2018.

Year	Herds	Animal Category	Heads	Mean (Standard Error)	Median (Range)
2008	931	Cows	20,086	21.6 (0.63)	15 (2–142)
		Heifers/beef	8752	9.4 (0.31)	7 (0–83)
		Calves	2797	3.0 (0.11)	2 (0–36)
2009	926	Cows	20,391	22.0 (0.63)	16 (2–159)
		Heifers/beef	8895	9.6 (0.30)	7 (0–77)
		Calves	2702	2.9 (0.11)	2 (0–31)
2010	907	Cows	20,837	23.0 (0.67)	18 (2–184)
		Heifers/beef	8784	9.7 (0.31)	7 (0–89)
		Calves	2823	3.1 (0.12)	2 (0–36)
2011	861	Cows	20,361	23.6 (0.69)	18 (2–162)
		Heifers/beef	8729	10.1 (0.33)	7 (0–80)
		Calves	2675	3.1 (0.12)	2 (0–40)
2012	831	Cows	19,960	24.0 (0.69)	19 (2–124)
		Heifers/beef	8233	9.9 (0.31)	8 (0–73)
		Calves	2655	3.2 (0.11)	2 (0–26)
2013	814	Cows	19,732	24.2 (0.68)	19 (2–113)
		Heifers/beef	8243	10.1 (0.32)	8 (0–90)
		Calves	2601	3.2 (0.12)	2 (0–31)
2014	763	Cows	19,635	25.7 (0.74)	20 (2–131)
		Heifers/beef	8338	10.9 (0.36)	9 (0–99)
		Calves	2600	3.4 (0.12)	2 (0–34)
2015	741	Cows	19,375	26.1 (0.75)	21 (2–111)
		Heifers/beef	8349	11.3 (0.36)	9 (0–92)
		Calves	2677	3.6 (0.14)	3 (0–43)
2016	717	Cows	19,197	26.8 (0.78)	22 (2–112)
		Heifers/beef	8134	11.3 (0.37)	9 (0–99)
		Calves	2584	3.6 (0.13)	2 (0–29)
2017	699	Cows	19,123	27.4 (0.81)	22 (2–128)
		Heifers/beef	7715	11.0 (0.37)	8 (0–94)
		Calves	2580	3.7 (0.14)	3 (0–39)
2018	693	Cows	18,896	27.3 (0.78)	23 (2–130)
		Heifers/beef	7633	11.0 (0.38)	9 (0–94)
		Calves	2512	3.6 (0.13)	3 (0–33)
TOTAL	8883	Cows	217,593	24.5 (0.21)	18.8 (2–183.9)
		Heifers/beef	91,805	10.3 (0.10)	7.7 (0–98.9)
		Calves	29,204	3.3 (0.04)	2.3 (0–43.2)

**Table 2:** Annual descriptive statistics on antimicrobial use (AMU) in cattle herds included in the study expressed as DDDAit per biomass. The use of antimicrobials included in WHO’s Highest Priority Critically Important Antimicrobials list (polymyxins, quinolones, macrolides, third- and fourth-generation cephalosporins) was considered critical. AMU was measured as DDDAit per biomass and the mean was weighted on the farm biomass.

Year	Herds	Antimicrobial Use	Weighted Mean (Standard Error)	Median (Range)
2008	931	Total	3.59 (0.07)	3.36 (0.06–18.13)
		Critical ^1^	0.53 (0.03)	0.14 (0–14.04)
2009	926	Total	3.76 (0.08)	3.44 (0.11–22.59)
		Critical ^1^	0.51 (0.03)	0.17 (0–8.10)
2010	907	Total	3.83 (0.08)	3.48 (0.06–21.40)
		Critical ^1^	0.56 (0.03)	0.16 (0–10.45)
2011	861	Total	4.00 (0.08)	3.70 (0.09–17.96)
		Critical ^1^	0.55 (0.03)	0.15 (0–7.82)
2012	831	Total	3.74 (0.08)	3.48 (0.03–17.94)
		Critical ^1^	0.52 (0.03)	0.17 (0–5.83)
2013	814	Total	3.64 (0.08)	3.43 (0.07–20.44)
		Critical ^1^	0.52 (0.03)	0.21 (0–5.22)
2014	763	Total	3.75 (0.08)	3.48 (0.07–18.80)
		Critical ^1^	0.56 (0.03)	0.19 (0–5.64)
2015	741	Total	3.40 (0.08)	3.16 (0.04–19.55)
		Critical ^1^	0.57 (0.03)	0.25 (0–19.55)
2016	717	Total	3.21 (0.08)	2.98 (0.03–14.52)
		Critical ^1^	0.48 (0.03)	0.18 (0–7.98)
2017	699	Total	3.32 (0.08)	3.28 (0.05–18.40)
		Critical ^1^	0.48 (0.03)	0.18 (0–6.08)
2018	693	Total	3.41 (0.08)	3.22 (0.07–20.27)
		Critical ^1^	0.49 (0.03)	0.19 (0–7.04)
TOTAL	8883	Total	3.61 (0.02)	3.35 (0.03–22.59)
		Critical^1^	0.52 (0.01)	0.19 (0–19.55)

^1^ WHO’s Highest Priority Critically Important Antimicrobials list (polymyxins, quinolones, macrolides, third- and fourth-generation cephalosporins).

**Table 3:** Distribution of antimicrobial classes used on the cattle population of the Aosta Valley (Italy) by year. Data for each class are expressed as percentage of total antimicrobial use (AMU). Classes included in the WHO’s Highest Priority Critically Important Antimicrobial list are highlighted in bold.

Antimicrobial Class	2008	2009	2010	2011	2012	2013	2014	2015	2016	2017	2018
Cephalosporins (1st and 2nd gen.)	4.59	6.20	6.97	7.48	10.86	13.11	12.15	13.44	14.25	16.10	17.35
Rifamycins	17.36	19.16	19.65	17.87	16.69	17.96	17.87	14.06	14.96	14.42	14.96
Penicillins Beta	18.07	13.20	13.95	14.03	12.10	10.08	12.54	12.51	12.75	12.53	13.36
Aminopenicillins	20.90	19.51	15.03	14.32	14.35	13.76	13.43	14.70	14.21	13.87	13.29
Aminoglycosides	10.10	13.16	14.57	16.92	16.44	15.20	13.43	13.48	13.83	13.49	12.77
**Cephalosporins (3rd and 4th gen.)**	**9.14**	**7.96**	**9.41**	**7.97**	**9.04**	**9.57**	**9.56**	**9.79**	**10.13**	**10.77**	**10.22**
Lincosamides	5.18	6.93	7.73	7.41	7.92	8.81	8.33	7.35	6.99	7.16	7.26
Penicillins	4.29	3.53	3.87	5.12	4.89	4.54	4.63	4.51	6.18	6.08	5.29
**Macrolides**	**4.47**	**4.39**	**3.99**	**4.44**	**3.82**	**3.45**	**3.93**	**4.55**	**3.44**	**2.83**	**3.19**
Tetracyclines	2.32	2.26	1.92	1.79	1.37	1.26	1.78	1.61	1.31	1.14	1.03
**Fluoroquinolones**	**1.08**	**1.08**	**1.07**	**1.16**	**0.91**	**1.08**	**1.11**	**1.88**	**1.06**	**0.99**	**0.78**
Sulfonamides	2.12	2.38	1.59	1.32	1.25	0.96	0.99	1.53	0.64	0.61	0.45
Amphenicols	0.20	0.17	0.14	0.10	0.26	0.14	0.02	0.04	0.02	<0.01	0.05
**Polymyxins**	**0.08**	**0.06**	**0.11**	**0.08**	**0.09**	**0.07**	**0.22**	**0.54**	**0.22**	**<0.01**	**<0.01**
Polypeptides	0.08	0	0.02	0	0	0	0	0	0	0	0
Tetracyclines	2.32	2.26	1.92	1.79	1.37	1.26	1.78	1.61	1.31	1.14	1.03

**Table 4:** Annual descriptive statistics on antimicrobial use (AMU) in dairy cows housed in the Aosta Valley herds. The use of antimicrobials included in WHO’s Highest Priority Critically Important Antimicrobials list (polymyxins, quinolones, macrolides, third- and fourth-generation cephalosporins) was considered critical. AMU was measured as a treatment incidence 100 (TI_100_) using daily dose animal for Italy (DDDAit) as metric. The mean was weighted on the standardised biomass of cows per herd.

Year	Herds	Antimicrobial Use (TI_100_) ^1^	Weighted Mean (Standard error)	Median (Range)
2008	931	Total	1.20 (0.02)	1.12 (0–5.26)
		Critical ^2^	0.16 (0.01)	0.02 (0–1.96)
2009	926	Total	1.25 (0.02)	1.12 (0–6.59)
		Critical ^2^	0.16 (0.01)	0.04 (0–2.28)
2010	907	Total	1.27 (0.03)	1.19 (0–7.42)
		Critical ^2^	0.18 (0.01)	0.05 (0–3.39)
2011	861	Total	1.33 (0.03)	1.23 (0–5.13)
		Critical ^2^	0.18 (0.01)	0.04 (0–2.71)
2012	831	Total	1.24 (0.03)	1.15 (0–5.91)
		Critical ^2^	0.17 (0.01)	0.04 (0–2.74)
2013	814	Total	1.20 (0.03)	1.15 (0–6.51)
		Critical ^2^	0.17 (0.01)	0.07 (0–1.74)
2014	763	Total	1.24 (0.03)	1.16 (0–6.01)
		Critical ^2^	0.18 (0.01)	0.06 (0–2.14)
2015	741	Total	1.09 (0.03)	0.99 (0–6.52)
		Critical ^2^	0.17 (0.01)	0.07 (0–6.52)
2016	717	Total	1.06 (0.03)	1.02 (0–4.71)
		Critical ^2^	0.15 (0.01)	0.06 (0–2.20)
2017	699	Total	1.24 (0.03)	1.21 (0–6.9)
		Critical ^2^	0.19 (0.01)	0.06 (0–2.28)
2018	693	Total	1.13 (0.03)	1.05 (0–5.8)
		Critical ^2^	0.16 (0.01)	0.06 (0–2.05)
TOTAL	8883	Total	1.21 (0.01)	1.12 (0–7.42)
		Critical ^2^	0.17 (0.01)	0.05 (0–6.52)

^1^ Treatment index 100. ^2^ WHO’s Highest Priority Critically Important Antimicrobials list (polymyxins, quinolones, macrolides, third- and fourth-generation cephalosporins).

## Data Availability

Considering that some of the data collected in this study were sensitive, data shall be available upon reasonable request and only after the anonymization of the herds and the medicinal products labels.
